# Case Report: Osimertinib Followed by Osimertinib Plus Bevacizumab, Personalized Treatment Strategy for a Lung Cancer Patient With a Novel *EGFR* Exon 20 Insertion D770_N771insGT and Multiple Brain Metastases

**DOI:** 10.3389/fonc.2021.733276

**Published:** 2021-10-25

**Authors:** Xiaoyu Zhi, Jiancheng Luo, Weiwei Li, Jinliang Wang, Yuying Wang, Yi Cai, Xiang Yan

**Affiliations:** ^1^ Department of Oncology, Chinese People’s Liberation Army (PLA) General Hospital, Beijing, China; ^2^ Medical School of Chinese People’s Liberation Army (PLA), Beijing, China; ^3^ Aiyi Technology Co., Ltd, Beijing, China; ^4^ Department of Oncology, The 81st Group Army Hospital of Chinese People’s Liberation Army (PLA), Zhangjiakou, China; ^5^ Independent Researcher, Ellicott City, MD, United States

**Keywords:** osimertinib, bevacizumab, exon 20 insertion mutation, non-small cell lung cancer, brain metastasis

## Abstract

Epidermal growth factor receptor (EGFR) tyrosine kinase inhibitors (EGFR-TKIs) are the standard of care for non–small cell lung cancer (NSCLC) patients with *EGFR* exon 19 deletion and L858R mutations. However, no EGFR TKI has been approved for NSCLC patients harboring insertion mutations in *EGFR* exon 20 (*EGFR*ex20ins), a subgroup of uncommon *EGFR* mutations resistant to first-generation EGFR TKIs. This unmet clinical challenge is further complicated by disease progression due to brain metastases (BMs), which limits the use of EGFR TKIs with low intracranial activity. Osimertinib, a third-generation EGFR TKI with high CNS activity, has demonstrated superior efficacy as a first-line treatment for *EGFR*-mutant NSCLC with or without BM. The VEGF pathway is a key mediator of cancer metastasis and resistance to EGFR TKIs. Accumulating evidence has demonstrated that the addition of anti-VEGF agents to EGFR TKIs provides an alternative treatment option for the clinical management of *EGFR*-mutant NSCLC. We herein report an NSCLC case with a novel *EGFR*ex20ins mutation D770_N771insGT and multiple brain metastases who briefly responded to first-line osimertinib treatment and subsequently achieved prolonged disease control with osimertinib plus bevacizumab as second-line treatment. Our case suggests that osimertinib in combination with bevacizumab may be an effective option for NSCLC patients with specific *EGFR*ex20ins mutations and brain metastases.

## Introduction

Epidermal growth factor receptor (*EGFR*) mutations are major drivers of non–small cell lung cancer (NSCLC) (1). Common *EGFR* mutations (exon 19 deletion and L858R) and some uncommon mutations (G719X, S768I, L861Q) are sensitive to EFGR tyrosine kinase inhibitors (TKIs) (2). In contrast, *EGFR* exon 20 insertion (*EGFR*ex20ins) mutations represent a heterogeneous group of uncommon *EGFR* mutations generally associated with a lack of response to EFGR TKIs (2).

The molecular heterogeneity of *EGFR*ex20ins mutations is largely mediated by three elements: position, length, and the exact amino acid sequence of the insertion (3, 4). Approximately 87% of *EGFR*ex20ins mutations are clustered in a span of 14 amino acids containing two motifs: the αC-helix (E762_M766) and the αC-β4 loop (A767_C775) (4). About 5% of *EGFR*ex20ins insertions are located at the N-terminus of the αC-helix (A763_Y764insX), which is sensitive to EGFR TKIs. In contrast, *EGFR*ex20ins mutants with insertions in the αC-β4 loop have an unaltered ATP-binding pocket and are intrinsically resistant to first-generation EGFR TKIs. In 2017, Kohsaka et al. developed a mixed-all-nominated-mutants-in-one (MANO) method to evaluate the drug sensitivity of 101 *EGFR* mutants to five EGFR TKIs (gefitinib, erlotinib, afatinib, osimertinib, and rociletinib). This work showed that five *EGFR*ex20ins mutants included in this study (S768_D770dup/D770_N771insSVD, N771_P772insN, H773_V774insH, H773_V774insPH, V774_C775insHV) are resistant to gefitinib, erlotinib, and rociletinib but display some varied sensitivity to afatinib and osimertinib (5). Furthermore, there was significant variation in the drug sensitivity of these five mutants as N771_P772insN was much more sensitive to afatinib/osimertinib than the others.

When EGFR TKIs are considered a treatment option for *EGFR*ex20ins-mutant NSCLC, physicians need to find the right EGFR TKI with high activity for *EGFR* mutants and selectivity over wild-type (WT) EGFR. A recent study showed that mobocertinib (TAK-788) inhibits the 4 most common *EGFR*ex20ins mutants (A767_V769dup, S768_D770dup, D770_N771insNPG, and N771_ H773dup) more potently than WT EGFR while afatinib showed the opposite pattern (6). Interestingly, osimertinib has selectivity for D770_N771insNPG but not the others. Given the heterogeneity of *EGFR*ex20ins mutations, the practice of personalized therapy in patients with these mutations requires a detailed analysis of specific variants.

The VEGF pathway is a key regulator of cancer metastasis and anti-VEGF therapies have been approved for use in NSCLC (7). Preclinical studies have found that VEGF and EGFR pathways share common downstream signaling, and their crosstalk can promote disease progression (8, 9). Recent clinical trials have demonstrated that the addition of anti-VEGF therapy to erlotinib in treatment-naive patients with *EGFR*-mutant NSCLC significantly improved their clinical outcomes (10, 11). Based on these results, the NCCN guidelines include erlotinib plus bevacizumab or ramucirumab as first-line treatment options for NSCLC patients with common *EGFR* mutations (2). However, whether EGFR/VEGF dual blockade could improve the clinical outcome of *EGFR*ex20ins-mutant NSCLC patients is unknown. Here, we present an NSCLC patient with a novel *EGFR*ex20ins mutation and brain metastases who achieved durable disease control with osimertinib plus bevacizumab after a brief response to first-line osimertinib monotherapy.

## Case Description

**Figure 1:**
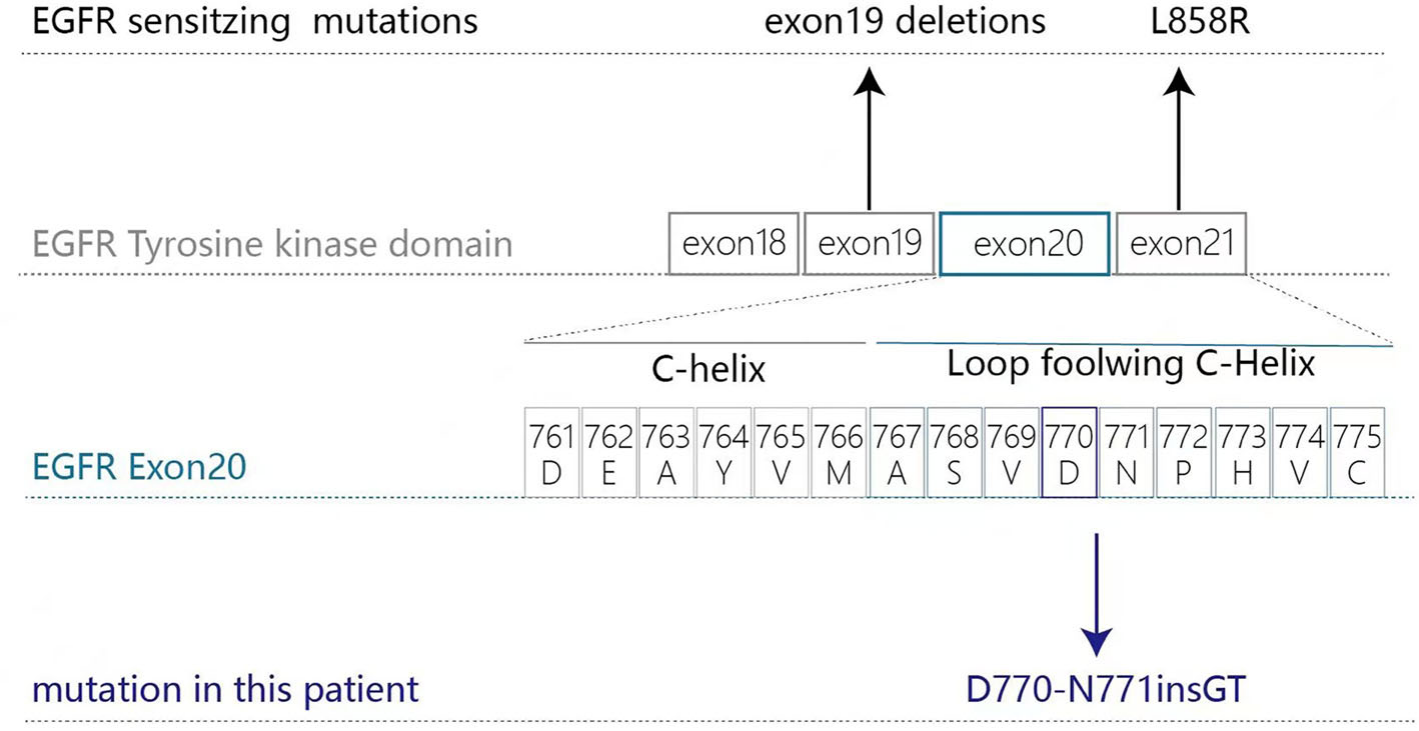
Gene check analysis showed that the patient had an *EGFR* exon20 insertion (p.D770-N771insGT) mutation.

In January 2019 a 69-year-old Chinese male ex-smoker who had had type 2 diabetes for 10 years presented with dizziness and unsteady walking for two months. Computed tomography (CT) scans of the chest revealed two masses and multiple nodules in the right lower lobe. Brain magnetic resonance (MR) imaging found multiple metastases and ECT showed enhanced radioactivity at T2 and L4. The patient was diagnosed with stage IV (T3N0M1) lung adenocarcinoma *via* percutaneous biopsy. Next-generation sequencing (NGS) testing showed an *EGFR* exon20 insertion (p.D770-N771insGT) mutation with concurring *ERBB2* and *TP53* mutations (**Figure 1**). According to the American College of Medical Genetics and Genomics (ACMG) guidelines (12), the *ERBB2* P1170A and *TP53* R197_V197insA mutations were classified as benign and a variant of unknown significance (VUS), respectively. Therefore, they were excluded from the treatment decision-making process.

**Figure 2:**
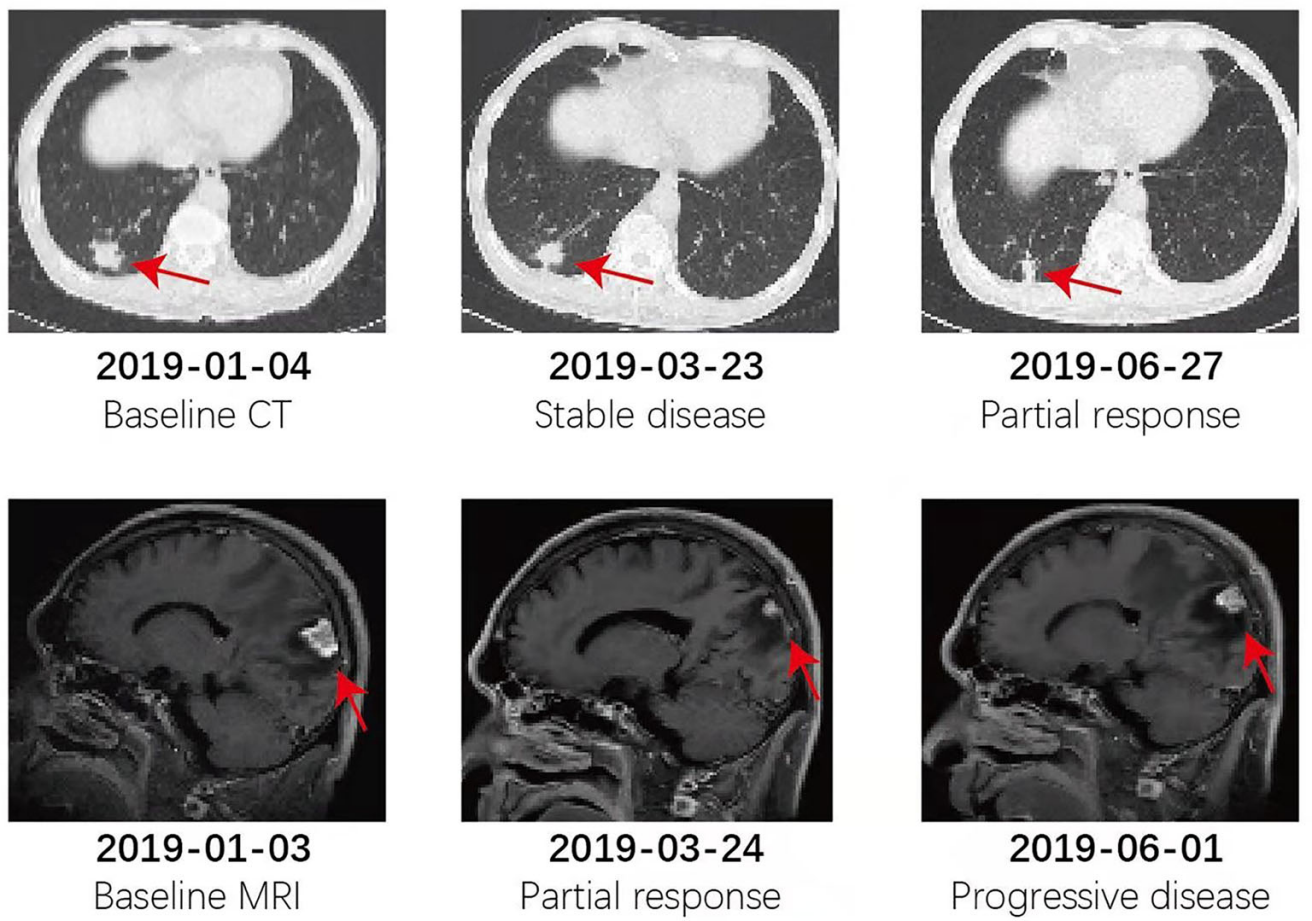
Patient dynamic changes in the chest CT and brain MRI during treatments.

The patient refused chemotherapy and whole-brain radiation, the standard care for his condition. As D770_N771insGT is a novel *EGFR*ex20ins mutant, we used data from two very similar mutants as the reference to guide our EGFR TKI selection process. For the D770_N771insNPG mutant, the IC50 values of erlotinib, afatinib, and osimertinib were 1146 nM, 43 nM, and 42 nM, respectively (13). The selectivity indexes (log10[IC50mutant/IC50Wild Type]) for afatinib and osimertinib were above zero and below -1, which means osimertinib has a better therapeutic dose window than afatinib for this variant. Importantly, Piotrowska et al. reported an 11- month-long clinical response to osimertinib in a metastatic lung cancer patient harboring an *EGFR* S768_D770dup mutation (14). Additionally, preclinical comparison of osimertinib with other EGFR TKIs in an *EGFR*-mutant NSCLC brain metastasis model showed that osimertinib has greater penetration of the blood-brain barrier than gefitinib, rociletinib, or afatinib at clinically relevant doses (15). The brain:plasma Cmax ratios for osimertinib, afatinib, gefitinib, and rociletinib were 3.41, <0.36, 0.21, and <0.08, respectively. Based on all these data, the patient was administered osimertinib (80 mg daily). It was well tolerated with only grade 1 nausea and his symptoms disappeared 20 days later. At the 4 month follow-up, the lesions on the right lung and brain achieved partial response by RECIST (version 1.1; −37.5% and -52.3% response, respectively). At the end of May, the patient complained about headache, and progression of the brain lesion was noted by MRI. With the consent of the patient, bevacizumab (400 mg/month) was added to osimertinib from June 6^th^, 2019. The combination was well tolerated without any report of toxicity. CNS symptoms cleared up again as the brain nodule stopped growing and edema was eliminated. Strikingly, the lung lesion achieved PR again (version 1.1; −82.2% response) with the combination therapy. At follow-up 2.1, 4.5, 6.5, and 9.1 months after the combination therapy, the patient had an ongoing clinical benefit and stable disease. The dynamic changes in chest CT and brain MRI during treatments are shown in **Figure 2**.

**Figure 3:**
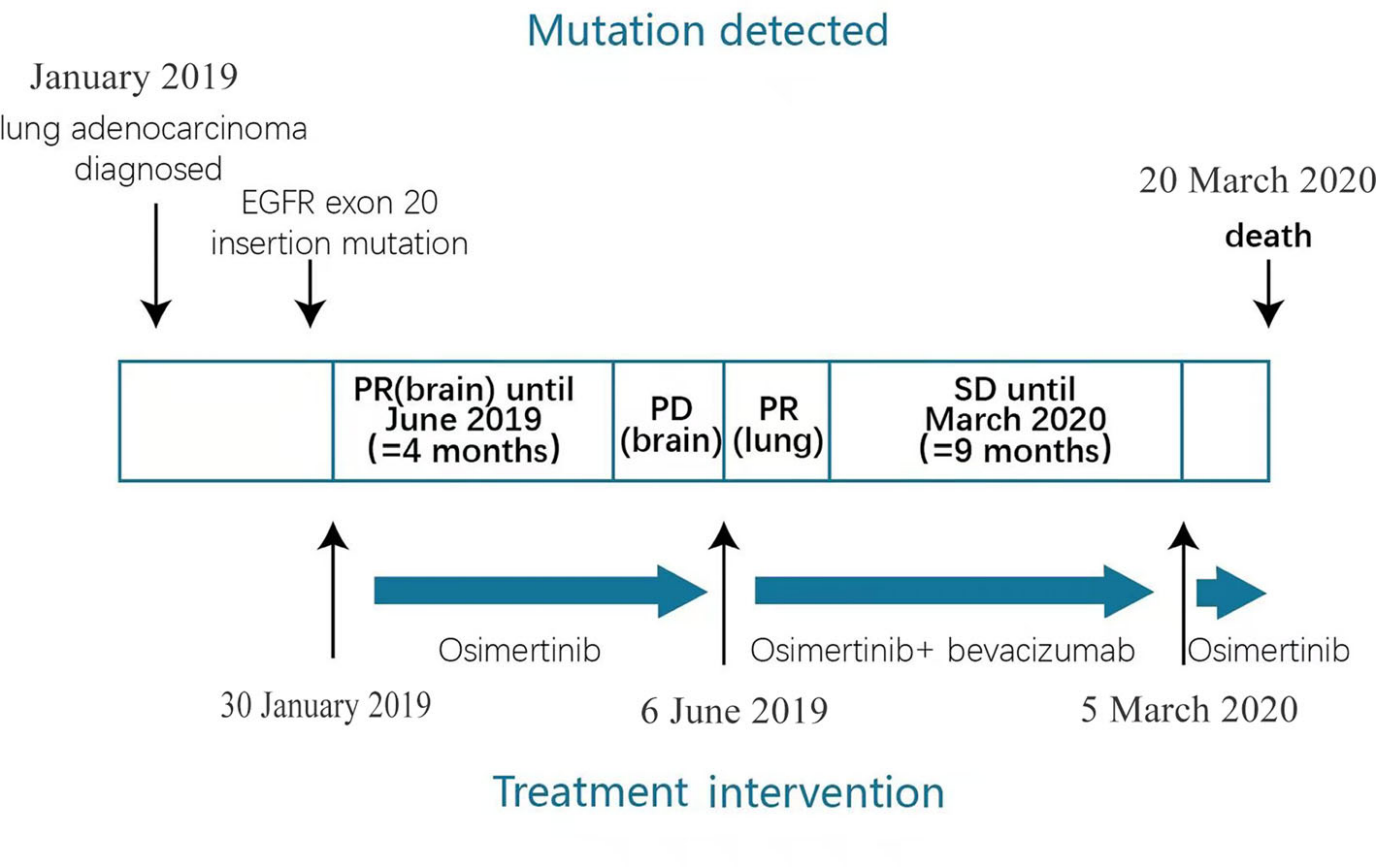
Patient clinical course including treatment process and relevant assessment.

Two months before the patient’s death, there was a significant decline in his physical activity and consciousness. On March 5th, a CT scan of the chest revealed no progressive disease. Brain MRI was not feasible due to his poor physical condition. Laboratory tests showed hemoglobin 96 g/L(normal range, 137-179 g/L), creatinine 177.7 umol/L (normal range, 30-110 umol/L), brain natriuretic peptide precursor (proBNP) 234.9 pg/mL (normal range, 0-150 pg/mL), carcinoembryonic antigen (CEA)22.46 ug/L (normal range, 0-5.0 ug/L), CYFRA21-1 5.15 ng/mL (normal range, 0.1-4.0 ng/mL), and SCC 3.1 ng/mL. Bevacizumab was discontinued due to the increased creatine level, a biomarker for kidney damage. Elevated proBNP levels are a heart failure biomarker related to cardiac adverse events associated with osimertinib. Because of his physical condition and the lack of symptoms of heart failure, the patient continued with osimertinib. He was discharged after his symptoms improved. Although we suspected the presence of leptomeningeal metastasis, we could not confirm this diagnosis with enhanced brain MRI or physical examination. Due to poor physical condition, the patient declined lumbar puncture for further diagnosis. Therefore, the exact cause of his death is unknown. On March 20th, the patient died with an overall survival time of 13.5 months. **Figure 3** shows a flow pathway for treatment and assessment.

## Discussion

Activating *EGFR* mutations are common drivers of NSCLC (1). *EGFR* exon 19 deletions and the L858R mutation represent 85-90% of kinase domain mutations observed in NSCLC and are sensitive to EGFR tyrosine kinase inhibitors (TKIs) (16). In contrast, uncommon *EGFR*ex20ins mutations are generally resistant to early-generation EGFR TKIs (17). Currently, no EGFR TKI has been approved for *EGFR*ex20ins-driven NSCLC and the standard of care for this condition is chemotherapy associated with 50-63% response rates and median PFS 4.1–6.4 months (18–20). Therefore, there is a substantial clinical need to improve the outcome of this NSCLC patient subgroup.

Currently, osimertinib is the preferred first-line EGFR TKI for NSCLC patients with sensitizing *EGFR* mutations (2). However, its efficacy in *EGFR*ex20ins NSCLC has not been adequately characterized. EA5162, a single-arm, phase 2 study showed that osimertinib at 160 mg achieved a response rate of 25%, disease control rate of 85%, and mPFS of 9.7 months in 20 *EGFR*ex20ins NSCLC patients (21). In a single-center retrospective study of osimertinib for 62 *EGFR*ex20ins NSCLC patients, osimertinib 80 mg or 160 mg resulted in a response rate of 6.5%, disease control rate of 53.2%, and mPFS of 2.3 months (22). There was no significant difference in median PFS between patients who received osimertinib 80 mg or 160 mg (2.5 *vs*. 1.3 months, P = 0.161).

Because *EGFR*ex20ins mutants are heterogeneous in their drug sensitivity, the selection of EGFR TKIs targeting specific *EGFR*ex20ins mutants requires a good understanding of their mechanism of activation and effects on drug binding. For instance, steric hindrance in the drug-binding pocket of some *EGFR*ex20ins mutants restricts the binding of EGFR TKIs with a rigid core and large terminal group such as osimertinib (6). Under this condition, afatinib can have a better inhibition potential than osimertinib. Consistently, the average IC50 values for afatinib and osimertinib across 6 *EGFR*ex20ins mutants (A767insASV, S768dupSVD, V769insASV, D770insNPG, D770insSVD, H773insNPH) were 39.9 nM and 103 nM, respectively (23). And afatinib achieved a durable response in a small number of NSCLC patients harboring specific *EGFR*ex20ins mutations (24–27). Similarly, poziotinib has a less rigid core and smaller terminal groups than osimertinib, which allows it to assess the restricted drug-binding pocket of *EGFR*ex20ins mutants more easily (16). In the ZENITH20-3 phase 2 trial, *EGFR*ex20ins NSCLC patients receiving poziotinib had an ORR of 27.8% (95% confidence interval [CI] 18.4–39.1%) and a median PFS of 7.2 months (28). Mobocertinib (TAK-788) is a novel EGFR TKI rationally designed to fit the drug-binding pocket of *EGFR*ex20ins mutants which results in increased affinity compared with osimertinib (6). In a phase 1/2 trial, pretreated *EGFR*ex20ins NSCLC patients receiving mobocertinib achieved an ORR of 28% and a median PFS of 7.3 months (29). Interestingly, amivantamab, an EGFR-MET bispecific antibody, was recently approved to treat *EGFR*ex20ins NSCLC patients (30). This approval was based on the results of a phase 1 trial CHRYSALIS (NCT02609776) in which amivantamab achieved an ORR of 40% (95% CI: 29%, 51%) and a median response duration of 11.1 months (95% CI: 6.9, not evaluable) (31).

Preclinical studies have found that the crosstalk between the VEGF and EGFR pathways plays an important role in the pathogenesis and metastasis of *EGFR*-mutant NSCLC (8, 9). Activation of EGFR signaling increases *VEGF* expression through hypoxia-independent mechanisms, and elevated VEGF, in turn, contributes to resistance to EGFR TKIs (9). Therefore, dual EGFR-VEGF inhibition may provide greater antitumor activity than the respective monotherapies (7). Indeed, the NEJ026 and ARTEMIS-CTONG1509 trials showed that PFS was significantly improved in the erlotinib plus bevacizumab group compared with the erlotinib group (16.9 *versus* 13.3 mo, HR: 0.605, p = 0.016; 17.9 *vs*. 11.2 mo, HR: 0.55, p < 0.001, respectively) (10, 32). The RELAY trial evaluated the efficacy of erlotinib plus ramucirumab *versus* erlotinib plus placebo (11). PFS was superior in the combination group compared with the control group (median PFS: 19.4 *versus* 12.4 mo, HR: 0.59, p < 0.0001). Currently, the NSCLC NCCN guidelines include erlotinib plus bevacizumab or ramucirumab as first-line treatments for patients with sensitizing *EGFR* mutations (2).

NSCLC patients with acquired erlotinib-resistant *EGFR* T790M mutations were excluded from most of the clinical trials mentioned above. Based on the superior efficacy of osimertinib against the T790M mutation and positive results of the RELAY and NEJ026 trials, it is expected that osimertinib plus bevacizumab may achieve better clinical efficacy than osimertinib alone in T790M-positive NSCLC patients. However, data from the randomized WJOG 8715L phase 2 trial revealed that this combination did not improve PFS or OS in T790M-positive NSCLC (33).

Here, we conducted a three-step analysis to reconcile the conflicting results between the WJOG 8715L trial and previous erlotinib/bevacizumab combination trials. First, we separated OS results from PFS results. Since no OS benefit was seen in phase 3 trials for erlotinib plus bevacizumab in *EGFR*-mutant NSCLC (7), one should not expect osimertinib plus bevacizumab to achieve longer OS than osimertinib alone. Second, we separated the first-line setting from the second-line setting in the PFS analysis. In the WJOG 8715L trial, the median PFS of second-line bevacizumab plus osimertinib was numerically shorter than that of osimertinib alone (9.4 *vs*. 13.5 months, p = 0.2). However, in the first-line setting, this combination achieved a median PFS of 19 months in a phase 1/2 trial conducted at MSKCC, close to the median PFS of 18.9 months with that of osimertinib alone in the FLAURA trial (34, 35). It is noteworthy that more patients in the MSKCC trial had brain metastases than those in the FLAURA trial (31% *vs.* 19%), which may influence PFS. Third, we identified multiple putative confounding factors in the WJOG 8715L trial. The percentages of stage IV diseases, prior chemotherapy, prior anti-VEGF therapy, and brain metastases in the combination group were all higher than those in the osimertinib group (83% *vs.* 63%, 25% *vs.* 17%, 20% *vs*. 10%, 30% *vs*. 22%) (33). Although these confounding factors were not statistically significant individually, all four factors were in favor of the osimertinib group. Furthermore, subgroup analysis revealed that patients with prior anti-VEGF therapy had a significantly shorter PFS than those who did not in the combination group (4.6 *vs.* 11.1 months; HR, 0.41; *P* = .03) but not in the osimertinib group (15.1 months *vs.* 13.7 months; HR, 1.19; *P* = .85) (33). Given the relatively small sample size (n = 81) and imbalanced patient characteristics of the WJOG 8715L trial, it will be too early to draw conclusions regarding the efficacy of osimertinib plus bevacizumab before the results of more trials become available.

In line with our analysis, the results of the ETOP BOOSTER phase 2 trial with a setting identical to the WJOG 8715L trial and larger sample size (n = 155) revealed that osimertinib plus bevacizumab yielded significantly longer median PFS in current/former smokers than osimertinib alone (16.5 *vs*. 8.4 months, p = 0.0052) (36). More importantly, the combination also achieved significantly longer median OS than osimertinib alone in the smoker subgroup. These results indicate that osimertinib plus bevacizumab could benefit the smoker subgroup of *EGFR*-mutant NSCLC patients.

One unresolved question is whether *EGFR*ex20ins-driven NSCLC will share similar downstream signaling profiles and vulnerabilities that can be exploited therapeutically with dual inhibition of EGFR and VEGF signaling. Interestingly, a preclinical study showed that three representative *EGFR*-activating mutations (exon 19 deletion, L858R, N771delinsFH) induce HIF-1α expression, which in turn upregulates *VEGF* expression (9). Osimertinib treatment reduced HIF-1α and VEGF in L858R or exon 19 deletion mutant cells while poziotinib treatment decreased HIF-1α and VEGF levels in N771delinsFH mutant cells. These results indicated that abnormal EGFR signaling activation was the dominant driver of VEGF upregulation in NSCLC cells and that EGFR-dependent upregulation of VEGF can be targeted with EGFR TKIs. Furthermore, in two PDXs harboring *EGFR*ex20ins mutations (H773insNPH and S768dupSVD), poziotinib plus bevacizumab resulted in near-complete tumor regression and significantly prolonged PFS compared with either reagent alone (9). These results indicated that the addition of bevacizumab to EGFR TKIs may achieve better therapeutic efficacy in *EGFR*ex20ins NSCLC than EGFR TKIs alone.

Accumulating evidence suggests that EGFR TKIs plus bevacizumab are a promising treatment option for *EGFR*-mutant NSCLC patients with CNS diseases. Data from the ARTEMIS-CTONG1509 phase 3 trial showed that the addition of bevacizumab to erlotinib significantly improved PFS in patients with brain metastases (32). Compared to erlotinib alone, the combination also had a positive trend to prolong OS in this patient subgroup. Similarly, in the MSKCC phase 2 trial, osimertinib plus bevacizumab resulted in a 100% response rate in 6 patients with brain metastases, including two complete responses (35). Interestingly, this combination also demonstrated CNS activity in patients with leptomeningeal metastases (37, 38). Consistently, the addition of bevacizumab to osimertinib achieved significantly longer PFS than osimertinib alone in our patient, who had multiple brain metastases.

## Conclusions

Our case suggests that osimertinib alone or in combination with bevacizumab can be a feasible therapeutic option for NSCLC patients with specific *EGFR*ex20ins mutations and brain metastases. Future studies are required to determine the efficacy and safety of this combination in NSCLC patients with different *EGFR*ex20ins mutations.

## Data Availability Statement

The original contributions presented in the study are included in the article/supplementary files. Further inquiries can be directed to the corresponding authors.

## Ethics Statement

Ethical review and approval was not required for the study on human participants in accordance with the local legislation and institutional requirements. The patients/participants provided their written informed consent to participate in this study. Written informed consent was obtained from the individual(s) for the publication of any potentially identifiable images or data included in this article.

## Author Contributions

Conception and design: XY, YC. Provision of study material or patients: XZ, XY, WL, JW. Collection and assembly of data: XZ, JL. Data analysis and interpretation: XZ, JL. Manuscript writing: All authors, mainly by YC. All authors contributed to the article and approved the submitted version.

## Funding

This study was supported by the National Natural Science Foundation of China (grant number 81902910).

## Conflict of Interest

Author JL was employed by company Aiyi Technology Co., Ltd.

The remaining authors declare that the research was conducted in the absence of any commercial or financial relationships that could be construed as a potential conflict of interest.

## Publisher’s Note

All claims expressed in this article are solely those of the authors and do not necessarily represent those of their affiliated organizations, or those of the publisher, the editors and the reviewers. Any product that may be evaluated in this article, or claim that may be made by its manufacturer, is not guaranteed or endorsed by the publisher.
