# Supplementary material for: Case Report: Osimertinib Followed by Osimertinib Plus Bevacizumab, Personalized Treatment Strategy for a Lung Cancer Patient With a Novel EGFR Exon 20 Insertion D770_N771insGT and Multiple Brain Metastases
Source: Front Oncol. 2021 Oct 25;11:733276. doi: 10.3389/fonc.2021.733276 (PMC8573166; doi:10.3389/fonc.2021.733276)
Supplement: Supplementary file 1 [file DataSheet_1.pdf]

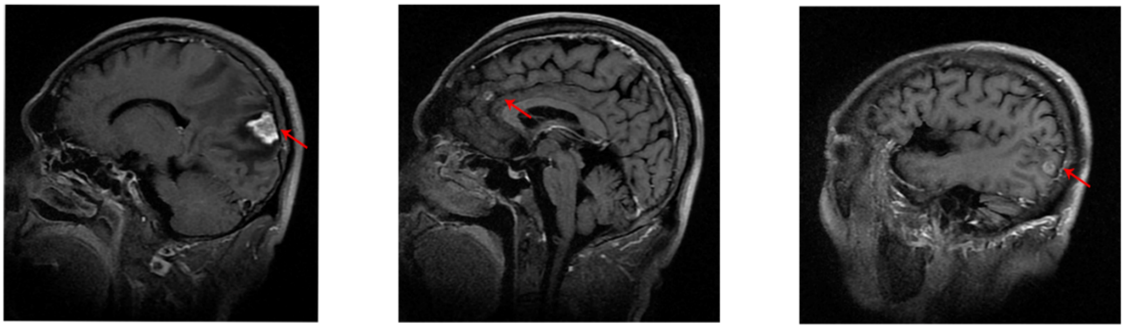

Supplementary Figure 1| Baseline MRI (2019-01-03)

The base line brain magnetic resonance imaging suggested multiple metastases, among which the biggest one was 2.1cm*2.4cm*1.3cm, located at the right parietal and occipital lobe junction (Supplementary Figure 1). During the treatment, only the right parietal and occipital lobe junction lesion shrank, while the other ones remained stable. We listed the size of the change in Figure 2. The patient's symptoms disappeared after osimertinib treatment, so he refused whole-brain radiation.

The patient presented a significant decline in physical activity and consciousness 2 months before the death, we considered that might be caused by meninges metastatic, even if neither the enhanced brain MRI nor the physical examination showed no positive findings. We discussed with the family about the necessary of lumbar puncture which helps a lot for both diagnosis and treatment. Due to poor physical activity, the patient was not able to cooperate with lumbar puncture, so the clinical speculation could not be confirmed.
